# Hydrogenase Gene Distribution and H_2_ Consumption Ability within the *Thiomicrospira* Lineage

**DOI:** 10.3389/fmicb.2016.00099

**Published:** 2016-02-08

**Authors:** Moritz Hansen, Mirjam Perner

**Affiliations:** Molecular Biology of Microbial Consortia, Biocenter Klein Flottbek, University of HamburgHamburg, Germany

**Keywords:** [NiFe]-hydrogenases, hydrogen consumption, *Thiomicrospira*, horizontal gene transfer, biogeography

## Abstract

*Thiomicrospira* were originally characterized as sulfur-oxidizing chemolithoautotrophs. Attempts to grow them on hydrogen failed for many years. Only recently we demonstrated hydrogen consumption among two of three tested *Thiomicrospira* and posited that hydrogen consumption may be more widespread among *Thiomicrospira* than previously assumed. Here, we investigate and compare the hydrogen consumption ability and the presence of group 1 [NiFe]-hydrogenase genes (enzyme catalyzes H_2_↔2H^+^ + 2e^-^) for sixteen different *Thiomicrospira* species. Seven of these *Thiomicrospira* species encoded group 1 [NiFe]-hydrogenase genes and five of these species could also consume hydrogen. All *Thiomicrospira* species exhibiting hydrogen consumption were from hydrothermal vents along the Mid-Atlantic ridge or Eastern Pacific ridges. The tested *Thiomicrospira* from Mediterranean and Western Pacific vents could not consume hydrogen. The [NiFe]-hydrogenase genes were categorized into two clusters: those resembling the hydrogenase from *Hydrogenovibrio* are in cluster I and are related to those from *Alpha*- and other *Gammaproteobacteria*. In cluster II, hydrogenases found exclusively in *Thiomicrospira crunogena* strains are combined and form a monophyletic group with those from *Epsilonproteobacteria* suggesting they were acquired through horizontal gene transfer. Hydrogen consumption appears to be common among some *Thiomicrospira*, given that five of the tested sixteen strains carried this trait. The hydrogen consumption ability expands their competitiveness within an environment.

## Introduction

*Thiomicrospira pelophila* was the first *Thiomicrospira* to be isolated in 1972 from estuarine mud in the Dutch Wadden Sea and was described as a sulfur-oxidizing bacterium (Kuenen and Veldkamp, 1972). In the years to follow at least 20 different *Thiomicrospira* strains were isolated from multiple environments including hydrothermal vents, intertidal mud flats, marine Arctic sediments, marine microbial mats, lake sediments, and salt water springs (e.g., Jannasch et al., 1985; Brinkhoff and Muyzer, 1997; Brinkhoff et al., 1999a,b,c; Takai et al., 2004; Knittel et al., 2005). All these *Thiomicrospira* were characterized as sulfur-oxidizers that could use various reduced inorganic sulfur compounds, such as sulfide, thiosulfate, tetrathionate, and sulfur under aerobic and/or microaerobic conditions. Most of them were identified as autotrophic CO_2_ fixers with some having been suggested to be chemolithomixotrophs (Takai et al., 2004).

For many years attempts to grow *Thiomicrospira* species on hydrogen failed (Nishihara et al., 1991; Takai et al., 2004). This was although a strain classified as *Hydrogenovibrio marinus*, which, according to 16S rRNA genes groups with *Thiomicrospira*, can use hydrogen (Nishihara et al., 1998, 2001). It was not until the genome of *T. crunogena* XCL-2, isolated from the Galapagos rift vents in the Eastern Pacific (Ahmad et al., 1999), was sequenced and all genes required for a structural [NiFe]-hydrogenase (capable of catalyzing H_2_↔2H^+^ + 2e^-^) and its assembly and maturation were detected (Scott et al., 2006) that hydrogen oxidation was considered as an alternative energy generation pathway. Genome sequencing has also identified a [NiFe]-hydrogenase gene in *Thiomicrospira* sp. MA2-6, isolated from a vent on the Mid-Atlantic ridge (MAR; Bioproject: PRJNA234785, K. Scott). And only recently we were able to demonstrate that *Thiomicrospira* sp. SP-41 and *T. crunogena* TH-55 also encode a [NiFe]-hydrogenase gene (through PCR amplification), most similar to that of XCL-2 (Hansen and Perner, 2015). Besides [NiFe]-hydrogenases, no other hydrogenases have been recognized on the genomes of XCL-2 or MA2-6. While no other [NiFe]-hydrogenases have been identified on any of the other so far sequenced *Thiomicrospira* genomes (Bioprojects: PRJNA182451, PRJNA169748, PRJNA165231, PRJNA214437, PRJNA214438, PRJNA165233, PRJNA204054 and PRJNA234827, K. Scott), Greening et al. (2015) have grouped sequences from *T. chilensis*, *T. kuenenii*, *T. pelophila*, and *H. marinus*, so far annotated as hypothetical proteins, into the group A1 [FeFe]-hydrogenases. When comparing these proteins with those from protein data bases using BlastP, the closest annotated [FeFe]-hydrogenases are those from *Roseibacterium elongatum* and *Wenzhouxiangella marina* (70 and 68% amino acid sequence identity; Riedel et al., 2014; Lee et al., 2015). However, no biochemical data is available for these putative [FeFe]-hydrogenases. It therefore remains uncertain whether the hypothetical proteins from *Thiomicrospira* and *Hydrogenovibrio* are functional [FeFe]-hydrogenases.

All so far identified [NiFe]-hydrogenases from *Thiomicrospira* and *Hydrogenovibrio* are classified as group 1 [NiFe]-uptake hydrogenases, where the hydrogenase of XCL-2 belongs to group 1b and that from *Hydrogenovibrio* to group 1d (Greening et al., 2015). The group 1 of [NiFe]-hydrogenases comprises membrane bound and non-membrane bound hydrogenases commonly located in the periplasm (Schwartz et al., 2013; Shomura and Higuchi, 2013). SP-41 and TH-55, which have the respective [NiFe]-hydrogenase, exhibit hydrogen uptake activity in the membrane but not in the soluble fraction (Hansen and Perner, 2015). In contrast, we could not amplify a [NiFe]-hydrogenase gene or detect any hydrogen consumption under the provided conditions for *T. thermophila* (Hansen and Perner, 2015). Likewise, Takai et al. (2005) could not identify any hydrogen uptake activity in *T. thermophila*’s cell extracts. No other biochemical analysis of a hydrogenase from *Thiomicrospira* species is available.

Since two of the three *Thiomicrospira* species we tested recently could consume hydrogen, we proposed that hydrogen consumption may be more widespread throughout this lineage than previously assumed. Having this trait makes *Thiomicrospira* more competitive. It broadens its physiological spectrum by making hydrogen an additional usable inorganic electron donor for potentially generating energy. This enhances its chances of successfully inhabiting environments where reduced sulfur compound concentrations are low, but hydrogen is available.

Here we tested and compared in total sixteen phylogenetically diverging *Thiomicrospira* isolated from geographically distinct hydrothermal vents, intertidal mud flats and lake systems for the presence of group 1 [NiFe]-hydrogenase genes, hydrogen uptake activity and *in vivo* hydrogen consumption rates: seven were from deep sea hydrothermal vents, namely XCL-2 (Galapagos rift, East Pacific; Ahmad et al., 1999), MA2-6 (Snake Pit, northern MAR; Muyzer et al., 1995), MA-3 (Trans-Atlantic Geotraverse, TAG, northern MAR; Wirsen et al., 1998), L-12 (Galapagos rift, East Pacific; Ruby and Jannasch, 1982), TH-55 (East Pacific rise, East Pacific; Jannasch et al., 1985), SP-41 (Sisters Peak, southern MAR; Hansen and Perner, 2015) and *T. thermophila* (TOTO caldera, Western Pacific; Takai et al., 2004), one from a shallow vent, Milos-T1 (near Milos, Greece; Brinkhoff et al., 1999c), three from intertidal mud flats in Germany, i.e., JB-B2 (Brinkhoff and Muyzer, 1997), *T. kuenenii* and *T. frisia* (Brinkhoff et al., 1999b), one from the Dutch Wadden Sea, *T. pelophila* (Kuenen and Veldkamp, 1972), one from marine Arctic sediments, *T. arctica* (Knittel et al., 2005), one from a *Thioploca* mat off the coast of Chile, *T. chilensis* (Brinkhoff et al., 1999a), one from a microbial mat in the Solar lake, Egypt, SL-1 (Brinkhoff and Muyzer, 1997) and one from a salt water spring in Germany, Art-3 (Brinkhoff and Muyzer, 1997). We found that hydrogen oxidation can be found in different *Thiomicrospira* strains but that it appears to be limited to few *Thiomicrospira* lineages.

## Material and Methods

### Strains and Cultivation Conditions

The strains *T. kuenenii*, *T. arctica*, *T. pelophila, T. chilensis*, *T. frisia*, JB-B2, MA-3, L-12, MA2-6, SL-1, Milos-T1, Art-3, XCL-2, TH-55, and *T. thermophila* were obtained from the Deutsche Sammlung von Mikroorganismen und Zellkulturen (DSMZ, Braunschweig, Germany). L-12 was additionally kindly provided by Jan Küver. The strain SP-41was already cultivated in our laboratory (Hansen and Perner, 2015). The *Thiomicrospira* strains were routinely cultivated in the medium recommended by the DSMZ, namely *T. pelophila* medium (Tp medium, DSMZ medium 142), *T. psychrophila* medium (DSMZ medium 142a) for *T. arctica*, or in TASW medium (Dobrinski et al., 2005). *T. thermophila* was cultivated in DSMZ medium 1011 using a H_2_:CO_2_ (80:20, Westfalen AG, Germany) gas mix instead of a N_2_:CO_2_ (80:20, Westfalen AG) gas mix. All strains were grown at 28°C with shaking except *T. arctica* and *T. thermophila*. *T. arctica* was cultivated at 10°C without shaking, *T. thermophila* at 37°C with shaking. For growth experiments without hydrogen, the tested strains were cultivated as described for the consumption experiments (see below) but instead of using a hydrogen gas mix, a N_2_:CO_2_ (80:20) gas mix was used in the headspace followed by the addition of sterile air (final concentration of 1% O_2_ in the headspace). Further information about all media and conditions used are given in **Table 1** and in the Supplementary Material.

**Table 1:** Media and cultivation conditions used to grow and investigate the different *Thiomicrospira* species.

Medium	Cultivation vessel	Thiosulfate	Gas phase	H_2_	O_2_
**Standard cultivation**					
T. pelophila medium	Flask	20 mM	Air	/	21%
T. pelophila medium	Hungate tube	20 mM	Air	/	21%
T. psychrophila medium	Flask	20 mM	Air	/	21%
DSMZ medium 1011	Serum bottle	6 mM	H_2_:CO_2_ + O_2_	∼60%	∼4%
TASW medium	Flask	40 mM	Air	/	21%
**H_2_ consumption experiments**					
MJ-T medium	Serum bottle	0.6 mM	H_2_:CO_2_:O_2_:He	2%	1%
T. pelophila medium^∗^	Serum bottle	20 mM	H_2_:CO_2_:O_2_:He	2%	1%
T. pelophila medium^∗^	Serum bottle	20 mM	Air + H_2_	∼1.5%	∼21%
MJ-T medium	Serum bottle	4 mM	H_2_:CO_2_:O_2_:He	2%	1%
MJ medium	Serum bottle	/	H_2_:CO_2_:O_2_:He	2%	1%
MJ-C medium	Serum bottle	/	H_2_:CO_2_:O_2_:He	2%	1%
MJ-T medium	Serum bottle	0.6 mM	Air + H_2_	∼1.5%	∼21%
TASW medium^∗^	Serum bottle	40 mM	H_2_:CO_2_:O_2_:He	2%	1%
MJ-T medium	Serum bottle	0.6 mM	H_2_:CO_2_:O_2_	79%	1%
**Growth experiments without H_2_**					
MJ-T medium	Serum bottle	0.6 mM	N_2_:CO_2_ + O_2_	/	1%
**Hydrogenase activity experiments**					
MJ-T medium	Serum bottle	0.6 mM	H_2_:CO_2_:O_2_	80%	1%


*^∗^Ni and Fe concentrations were increased to be the same as in MJ-T medium (i.e., 3 μM Ni and 30 μM Fe).*

### *In Vivo* Hydrogen Consumption Measurements

Hydrogen consumption measurements were carried out as described before (Hansen and Perner, 2015). All strains were cultivated in MJ-T medium in serum bottles closed with rubber stoppers (for details, see Hansen and Perner, 2015). The headspace contained H_2_:CO_2_:O_2_:He (2:20:1:77, Westfalen AG). For the strains not growing in MJ-T medium, Tp medium with raised Ni and Fe concentrations (∼0.003 mM and 0.03 mM final concentration, respectively, same as in MJ-T medium) under the H_2_:CO_2_:O_2_:He (2:20:1:77) gas mix was tested. For the strains still not growing, Tp medium with increased Ni and Fe concentrations and an elevated oxygen concentration was tested, i.e., the medium was filled in serum bottles under air and then H_2_ (H_2_ 5.0, Westfalen AG) added to give a final hydrogen concentration in the headspace of ∼1.5%. XCL-2 was additionally tested in different media and under different conditions (see **Table 1** and Supplementary Material). All experiments were set up in triplicate and non-inoculated medium treated the same way as the samples were used as controls. Pre-cultures were setup also in MJ-T medium under the same conditions or in the media stated above. The samples for measurements were inoculated ∼1:100 with the pre-cultures in new serum bottles, the headspace gassed again with the gas mix and the starting hydrogen concentration measured immediately after. Hydrogen concentration was determined via gas chromatography (Trace GC Ultra gas chromatograph, Thermo Fisher Scientific Inc., Waltham, MA, USA) with a ShinCarbon ST 100/120 column (Restek Corporation, Bellafonte, PA, USA) and a Pulse Discharge Detector (Vici Valco Instruments, Houston, TX, USA). The carrier gas was He 5.0 (Linde Group, München, Germany). Cell numbers were counted directly using a counting chamber (Hawksley, Sussex, UK) and calculations were performed as before (Perner et al., 2010). At the end of the experiment, the cultures were checked for purity by amplification and sequencing of 16S rRNA genes and [NiFe]-hydrogenase genes (if present), respectively (see below).

### Hydrogenase Activity Measurements

The strains L-12, MA-3, MA2-6, XCL-2, and JB-B2 were tested for hydrogen uptake activity. They were cultivated in MJ-T medium under H_2_:CO_2_:O_2_ (79:20:1, Westfalen AG) for 2–3 days at 28°C and regassed. Extraction of different cell fractions and measurements of hydrogen uptake activity were performed as described before (Hansen and Perner, 2015 and references therein). Enzyme activities were determined spectrophotometrically with methyl viologen as artificial electron acceptor.

### DNA Extraction and PCR Conditions

DNA from the different strains was extracted using the Ultra clean microbial DNA isolation kit (Mo Bio Laboratories Inc., Carlsbad, CA, USA) according to the protocol provided by the manufacturer. 16S rRNA genes were amplified as described before (Perner et al., 2009) using the primers 27F and 1492R (Lane, 1991). PCR products were cleaned up using the Gel/PCR DNA Fragments Extraction Kit (Geneaid Biotech Ltd., New Taipei City, Taiwan). For double stranded sequencing of the 16S rRNA gene from L-12 the primers 27F, 1492R (Lane, 1991) as well as 562R, 719F and 1149R (Hansen and Perner, 2015) were used. All investigated *Thiomicrospira* species were tested for the presence of group 1 [NiFe]-hydrogenase genes using different primer sets. PCR conditions for the primer set hynL110F and hynL410R (Takai et al., 2005) was described before (Hansen and Perner, 2015). To amplify the group 1 [NiFe)-hydrogenase gene from MA2-6, the primers MA2-6hynLF (TTA TAT GGC GGC GGT CTA TC) and MA2-6hynLR (ATC ATC CCG CAT CAT GTC TC) were designed based on the respective hydrogenase gene sequence from MA2-6. PCR conditions were: initial denaturation at 95°C for 5 min followed by 32 cycles of denaturation at 95°C for 45 s, annealing at 55°C for 45 s, and elongation at 72°C for 45 s. To amplify group 1 [NiFe]-hydrogenase genes resembling SP-41’s and XCL-2’s, the degenerated primers TmshynL24F (GAA AAT YGT AAT YGA TCC RGT C) and TmshynL1669R (AGG TKT GRC CTT GCG CAT C) were designed from the respective aligned hydrogenase genes from SP-41 and XCL-2. PCR conditions were: initial denaturation at 95°C for 5 min followed by 32 cycles of denaturation at 95°C for 45 s, annealing at 49°C for 45 s and elongation at 72°C for 110 s. For sequencing of both strands the primers TmshynL24F and TmshynL1669R, hynL110F, and hynL410R (Takai et al., 2005) as well as the primers 90R, 208F, 338R, 614F, 656F, and 714R (Hansen and Perner, 2015) were used.

### Sequence Processing and Data Deposition

Sequences were analyzed and edited using Lasergene Software SeqMan (DNASTAR, Madison, WI, USA). Phylogenetic trees were constructed using Seaview (Gouy et al., 2010) with PhyML (Guindon and Gascuel, 2003) and 100 bootstraps. All new sequences obtained with this work, namely the [NiFe]-hydrogenase from MA-3, L-12, and JB-B2 as well as amended [NiFe]-hydrogenase sequence from TH-55 and 16S rRNA gene sequence from L-12 were deposited in the National Center for Biotechnology Information (NCBI) under the accession numbers KT717680-KT717684.

## Results and Discussion

**FIGURE 1:**
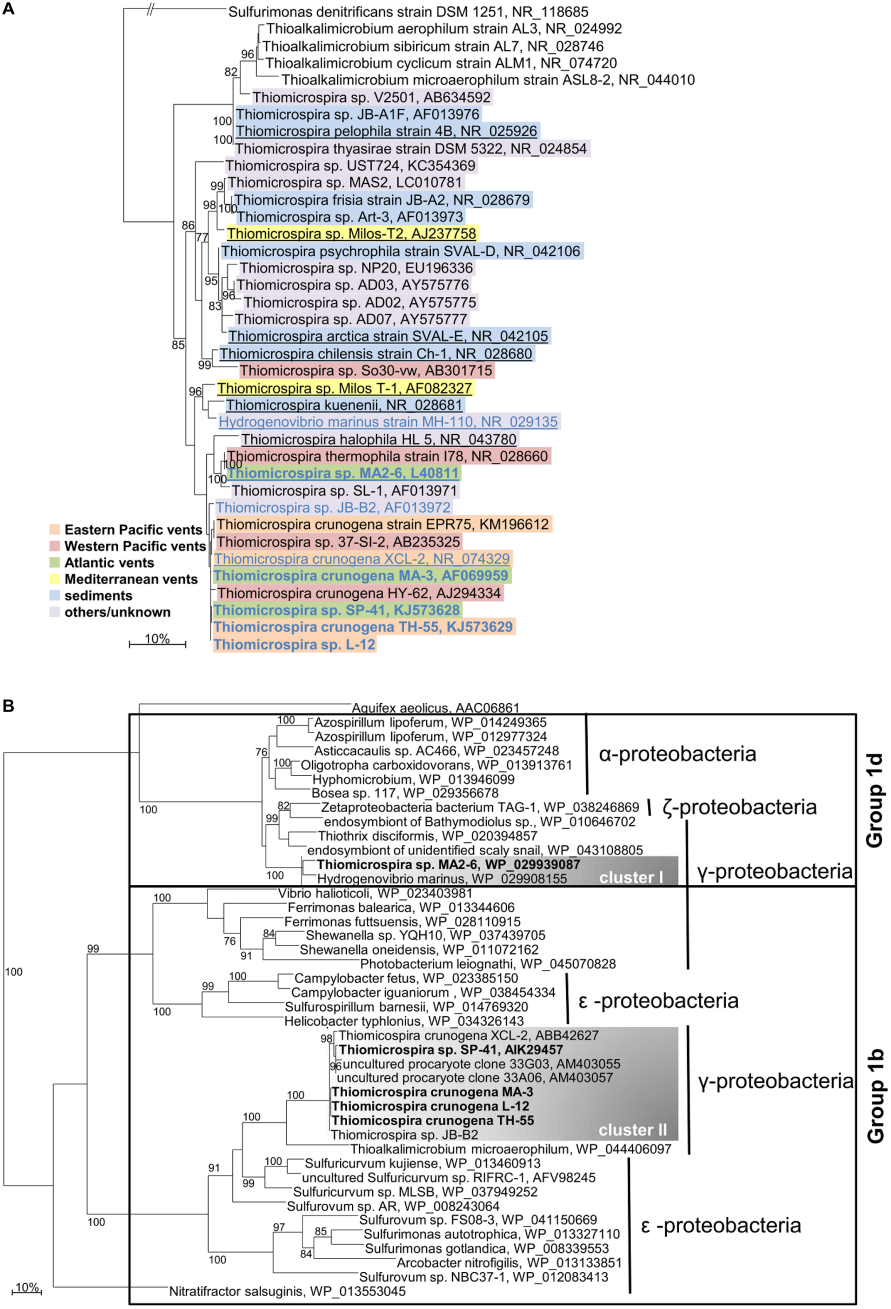
**Phylogenetic relationship of *Thiomicrospira* species.** **(A)** 16S rRNA genes. *Thiomicrospira* species are color-coded according to their isolation source. Species that are underlined indicate that genomic sequence data is available. Species for which a *hynL* gene is known are written in blue, those which consumed hydrogen in our experiments are written in bold. **(B)** [NiFe]-hydrogenase large subunit genes (*hynL)*. Species which consumed hydrogen in our experiments are written in bold. Only bootstrap values ≥ 75 are given. The scale bar represents the expected number of changes per **(A)** nucleotide and **(B)** amino acid. Classification in **(B)** is according to Greening et al. (2015). *Aquifex aeolicus’* [NiFe]-hydrogenase belongs to group 1e.

Based on 16S rRNA gene analyses the different *Thiomicrospira* species form a group together with *H. marinus* MH-110 (**Figure 1A**). Since *H. marinus* exhibits 98 and 97% 16S rRNA sequence identity to its closest relatives *Thiomicrospira* sp. Milos T-1 and *T. kuenenii*, respectively, this may call for a reclassification of this genus.

### Hydrogenase Phylogeny and Horizontal Gene Transfer

The large subunit of a group 1 [NiFe]-hydrogenase [here called *hynL* as proposed by Vignais et al. (2001) for a number of hydrogenase genes] has so far been identified in XCL-2 (Scott et al., 2006), TH-55, and SP-41 (Hansen and Perner, 2015), as well as MA2-6 (Bioproject: PRJNA234785, K. Scott; **Figure 1B**). In this study, we identified three new *hynL* gene sequences from the 12 further tested strains using three different primer sets targeting these group 1 [NiFe]-hydrogenase genes: one degenerated set to amplify *hynL* genes from a variety of different bacteria (Takai et al., 2005), one for *hynL* genes resembling those from XCL-2 and SP-41, and one specific for the *hynL* gene from MA2-6. The newly identified *hynL* genes were from MA-3, L-12, and JB-B2 (**Figure 1B**). According to the phylogeny of the now seven known *Thiomicrospira hynL* genes, two *hynL* gene clusters are evident: cluster I, within the group 1d, and cluster II, within the group 1b of the [NiFe]-hydrogenases [see **Figure 1B**, classification according to Greening et al. (2015)].

The cluster I includes the *hynL* genes from *H. marinus* and from MA2-6 (HynL amino acid sequence identity 92%; 16S rRNA gene identity 95%). Their [NiFe]-hydrogenases are closely related to those from *Alpha*-, *Gamma*- and *Zetaproteobacteria*. The hydrogenase from *H. marinus* belongs to the membrane-bound respiratory [NiFe]-hydrogenases (MBH) and is well studied: it is located in the membrane fraction (Nishihara et al., 1989, 1997, 2001), anchored in the membrane via a cytochrome b and oxidizes hydrogen in the periplasmic space (Shomura et al., 2011) and it is highly resistant toward inactivation by oxygen (Nishihara et al., 1997). From its crystal structure it was deduced that unlike standard [NiFe]-hydrogenases, the proximal [Fe-S]-cluster is a [4Fe-3S] type (commonly [4Fe-4S] type), is coordinated by six cysteine residues and mediates the oxygen stability of the [NiFe]-hydrogenase (Shomura et al., 2011). The genes for the large and small subunit incorporate the characteristic sequence motifs for the group 1d, namely the L1 and L2 motifs (Greening et al., 2015) and the binding motifs of the proximal, medial, and distal [Fe-S]-cluster (Pandelia et al., 2012; Greening et al., 2015). The [NiFe]-hydrogenase from MA2-6 has not been biochemically characterized. However, since its genome encodes a cytochrome b subunit, it can be expected that it is also membrane bound. MA2-6’s [NiFe]-hydrogenase small subunit has the specific binding motif for the untypical proximal [Fe-S]-cluster (Greening et al., 2015), which is responsible for the oxygen tolerance (Pandelia et al., 2012). Hence, MA2-6’s [NiFe]-hydrogenase may well also be oxygen tolerant.

All other identified *hynL* genes group into cluster II and are affiliated with *T. crunogena* and close relatives: these are SP-41, XCL-2, TH-55, MA-3, L-12, and JB-B2 (HynL amino acid sequence identity ≥97%; 16S rRNA gene identity ≥99%). Together with the *Gammaproteobacterium Thioalkalimicrobium microaerophilum* (HynL amino acid sequence identity ≤78%, 16S rRNA gene identity 91%), this clade forms a monophyletic group with *hynL* genes from *Epsilonproteobacteria.* Until now not much on these cluster II *Thiomicrospira* hydrogenases is known: Measurements of hydrogen uptake activity of SP-41 and TH-55 demonstrated that the active hydrogenases are located in the membrane fraction (Hansen and Perner, 2015). It was also shown that the group 1 [NiFe]-hydrogenase from SP-41 was transcribed and is thus likely the transcript encoding for the active hydrogenase (Hansen and Perner, 2015). Hydrogenases from MA-3 and L-12 are also active in the membrane fraction (detailed discussion below). While it can be debated that the hydrogen uptake activity in the *Thiomicrospira* species may not be related to the group 1 [NiFe]-hydrogenases but to other hydrogenases, we argue against this for three reasons: (i) the *hynL* gene was transcribed in hydrogen uptake active SP-41 cultures (Hansen and Perner, 2015), (ii) it appears highly unlikely that the group I [NiFe]-hydrogenases from the *Thiomicrospira* would be so conserved, at least for the cluster II hydrogenases, if they were not principally encoding functional hydrogenases (XCL-2 may not consume hydrogen and not exhibit hydrogen uptake activity for other reasons, see discussion below) and (iii) the only other so far potential hydrogenases that have been identified in *Thiomicrospira*, namely [FeFe]-hydrogenases in *T. chilensis, T. kuenenii*, and *T. pelophila* (Greening et al., 2015), do not appear to be hydrogen uptake active under the provided conditions, since we could not measure hydrogen consumption for these species in our cultures (detailed discussion below). For these reasons we think that it is highly likely that the group 1 [NiFe]-hydrogenases are involved in hydrogen consumption in *Thiomicrospira*.

According to the classification of Greening et al. (2015), the cluster II *Thiomicrospira* hydrogenases belong to the group 1b of the [NiFe]-hydrogenases, the prototypical hydrogenases. Well studied representative hydrogenases of this group are from *Wolinella succinogenes, Desulfovibrio gigas*, and *Helicobacter pylori*. The *W. succinogenes* hydrogenase is heterodimeric (30 and 60 kDa subunits), membrane anchored by a cytochrome b subunit and oriented toward the periplasm (Dross et al., 1992; Gross et al., 1998). *D. giga’s* hydrogenase is a periplasmic heterodimer (26 and 62 kDa subunits; Bell et al., 1974; Hatchikian et al., 1978) and *H. pylori’s* hydrogenase consists of a 26 kDa small subunit, a 65 kDa large subunit and is associated with the membrane (Maier et al., 1996). In contrast, XCL-2’s hydrogenase differs from these hydrogenases: the small subunit misses the characteristic Tat-signal (Scott et al., 2006), which is an important signal for the translocation of the enzyme from the cytoplasm (Vignais et al., 2001 and references therein). It is proposed that the enzyme is located in the cytoplasm (Scott et al., 2006). XCL-2 does not encode for a cytochrome b subunit, which commonly anchors [NiFe]-hydrogenases in the membrane (Vignais et al., 2001). Furthermore, the small subunit carries rather untypically a pyridine nucleotide-disulfide oxidoreductase with a NADH-binding site suggesting that it may react directly with its redox partner (Scott et al., 2006). With its length of 813 amino acids (estimated 90 kDa) it is much larger than the group 1 [NiFe]-hydrogenase small subunits usually are (Vignais and Billoud, 2007). However, since no genomic information of other *Thiomicrospira* encoding a cluster II hydrogenase is available, we cannot compare the small subunits with each other to test whether they all have this feature or whether this is a unique feature of XCL-2 among this lineage. As cluster II hydrogenases show only 40 to 41% amino acid identity to cluster I hydrogenases, these hydrogenases likely exhibit different characteristics.

The phylogenetic distribution and branching patterns of the *hynL* genes (**Figure 1B**) can be interpreted by distinct evolutionary scenarios: In one scenario the cluster I hydrogenases may be the original [NiFe]-hydrogenases that originate from a common ancestor and were lost in all other *Thiomicrospira* species. On one hand the relatively large 16S rRNA gene discrepancy between *H. marinus* and MA2-6 may support this. On the other hand it seems very unlikely that all of the other *Thiomicrospira* species have lost these genes, particularly since some strains colonize hydrothermal vents, where hydrogenases would be advantages. Alternatively, the cluster I hydrogenases may have been taken up by *H. marinus* and MA2-6 independently from each other, because the enzymes combine characteristics that are favorable in the environments the bacteria live. This would also explain the incongruence between *H. marinus’* and MA2-6’s [NiFe]-hydrogenase gene sequence, 16S rRNA phylogeny and geography if they both took this gene up. *H. marinus*’ hydrogenase is known to be extremely oxygen stable (Nishihara et al., 1997), which it needs to be given that *H. marinus* was isolated from seawater (Nishihara et al., 1989). MA2-6’s [NiFe]-hydrogenase may also be oxygen stable, given that the small subunit has the binding motif for the oxygen tolerance mediating [4Fe-3S]-cluster. If this were the case, oxygen stability may be a feature of cluster I hydrogenases. It would be interesting to find more *Thiomicrospira* species harboring a cluster I hydrogenase to see how closely related they are to MA2-6 and *H. marinus* and how the geographical distribution is.

Given that all of the tested *T. crunogena* species have a cluster II group 1 [NiFe]-hydrogenase and exhibit high *hynL* and 16S rRNA gene identities (**Figure 1**), we posit that a common ancestor of *T. crunogena* has taken up these [NiFe]-hydrogenase genes via horizontal gene transfer (HGT). Acquisition of distinct metabolic traits through HGT has been proposed for *Thiomicrospira* species before. For example, in XCL-2 a genome region containing a phosphonate operon appears to have been taken up (Scott et al., 2006) and *Thiomicrospira* sp. HY-62 likely acquired its sox enzyme system from an *Alphaproteobacterium* (Petri et al., 2001). In the Lost City *Thiomicrospira* population the sox system also appears to have been acquired via HGT and a high transposase content suggested HGT to play an important role during evolution of the Lost City *Thiomicrospira* population (Brazelton and Baross, 2010). Given that the cluster II hydrogenases form a monophyletic group with those from *Epsilonproteobacteria* and since *Thiomicrospira* and *Epsilonproteobacteria* colonize similar environments like hydrothermal vents (Ruby et al., 1981; Ruby and Jannasch, 1982; Campbell et al., 2006) or marine sediments (Timmer-Ten Hoor, 1975; Brinkhoff et al., 1999b), we posit that the cluster II hydrogenases were acquired by a *T. crunogena* common ancestor either from an *Epsilonproteobacterium* or from the same bacteria that *Epsilonproteobacterium* acquired the hydrogenase from. This theory is also in line with the absence of these [NiFe]-hydrogenase genes in all our other investigated *Thiomicrospira* species (see **Table 2** and Bioprojects PRJNA182451, PRJNA169748, PRJNA214437, PRJNA214438, and PRJNA204054, K. Scott) and in the additional three strains *T. halophila*, *Thiomicrospira* sp. KP2, and *Thiomicrospira* sp. Milos-T2 for which draft genome sequences are available (Bioprojects: PRJNA165231, PRJNA165233, PRJNA234827, K. Scott).

**Table 2:** Strains investigated in this study and *hynL* gene amplification results.

Strain	Isolation source	*hynL* PCR			H_2_ consumed	Isolation reference
				
		1	2	3		
*T.* sp. SP-41	Sisters Peak (MAR-S)	+	+	–	+	Hansen and Perner, 2015
*T.* sp. MA2-6	Snake Pit (MAR-N)	–	–	+	+	Muyzer et al., 1995
*T. crunogena* MA-3	TAG (MAR-N)	+	+	–	+	Wirsen et al., 1998
*T. crunogena* TH-55	East Pacific rise, EP	+	+	–	+	Jannasch et al., 1985
*T. crunogena* L-12	Galapagos rift, EP	+	+	–	+	Ruby and Jannasch, 1982
*T. crunogena* XCL-2	Galapagos rift, EP	+	+	–	–	Ahmad et al., 1999
*T. thermophila*	Mariana Arc, WP	–	–	–	–	Takai et al., 2004
*T.* sp. Milos-T1	Milos, Greece	–	–	–	–	Brinkhoff et al., 1999c
*T. pelophila*	Wadden Sea, Netherlands	–	–	–	–	Kuenen and Veldkamp, 1972
*T. kuenenii*	Jadebusen, Germany	–	–	–	–	Brinkhoff et al., 1999b
*T. frisia*	Jadebusen, Germany	–	–	–	–	Brinkhoff et al., 1999b
*T.* sp. JB-B2	Jadebusen, Germany	–	+	–	–	Brinkhoff and Muyzer, 1997
*T. arctica*	Arctic sediment, Norway	–	–	–	–	Knittel et al., 2005
*T*. sp. SL-1	Solar lake, Egypt	–	–	–	–	Brinkhoff and Muyzer, 1997
*T. chilensis*	Continental shelf, Chile	–	–	–	–	Brinkhoff et al., 1999a
*T*. sp. Art-3	Saline spring, Germany	–	–	–	–	Brinkhoff and Muyzer, 1997


*PCRs: 1: primer pair hynL110F and hynL410R (Takai et al., 2005); 2: TmshynL24F and TmshynL1669R; 3: MA2-6hynLF and MA2-6hynLR. MAR, Mid-Atlantic Ridge (South or North); EP, East Pacific; WP, West Pacific.*

### H_2_ Consumption and Hydrogen Uptake Activity

**FIGURE 2:**
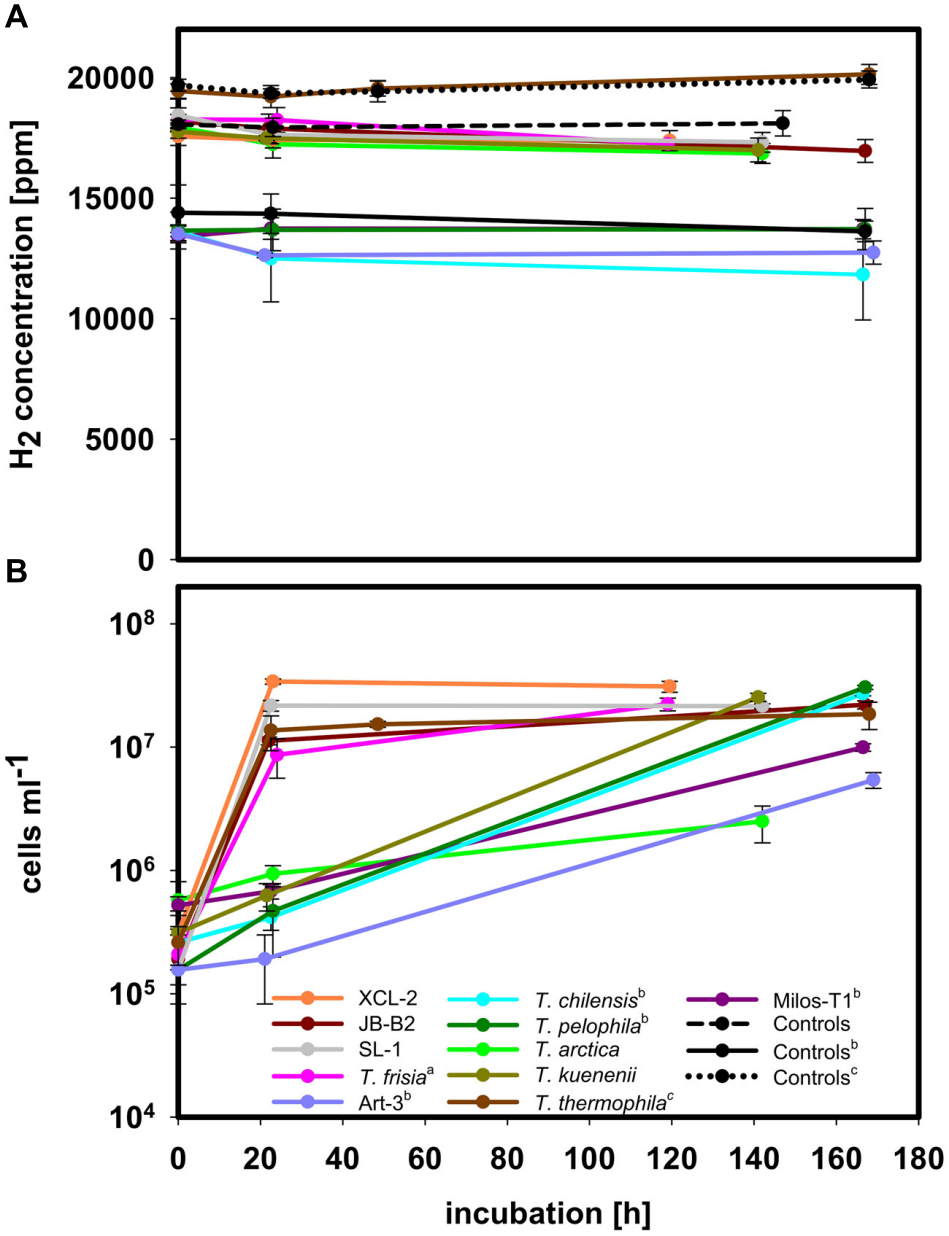
**H_2_ consumption experiments with species not consuming hydrogen under incubation conditions.** **(A)** H_2_ concentration in the headspace during incubation. **(B)** Cell density during incubation of the respective species. All experiments were performed at 28°C except for *T. arctica* and *T. thermophila*, which were undertaken at 10°C and 37°C, respectively. Experiments were performed in MJ-T medium under an atmosphere of H_2_:CO_2_:O_2_:He (2:20:1:77) or (^a^) in Tp medium with added Ni and Fe to give the respective concentrations as in MJ-T medium under an atmosphere of H_2_:CO_2_:O_2_:He (2:20:1:77) or (^b^) in Tp medium with Ni and Fe and in air with added H_2_ to give a final concentration of approximately 1.5% hydrogen. For the H_2_:CO_2_:O_2_:He gas mix two sets of controls, non-inoculated medium treated the same way as the samples, are shown as broken and dotted black line, respectively, and controls for the mixture of air and H_2_ one set of controls is shown. (^c^) Data from Hansen and Perner (2015).

*Thiomicrospira* strains for which we could not amplify a [NiFe]-hydrogenase gene successfully – using three different primer sets (**Table 2**) despite growth in the medium were: *T. thermophila* (data from Hansen and Perner, 2015), as well as *T. arctica*, *T. chilensis*, *T. frisia*, *T. kuenenii*, *T. pelophila*, SL-1, Art-3, and Milos-T1 (**Figure 2**; for more details see Supplementary Material results). These *Thiomicrospira* were isolated from a Western Pacific vent, sediments, a microbial mat, and a Mediterranean vent (Kuenen and Veldkamp, 1972; Brinkhoff and Muyzer, 1997; Brinkhoff et al., 1999a,b,c; Takai et al., 2004; Knittel et al., 2005). Although *T. chilensis*, *T. kuenenii*, and *T. pelophila* have been suggested to have [FeFe]-hydrogenases (Greening et al., 2015), they did not consume any hydrogen under the experimental conditions (**Figure 2**). All these *Thiomicrospira* do not appear to depend on hydrogen uptake activity under the provided incubation conditions.

**FIGURE 3:**
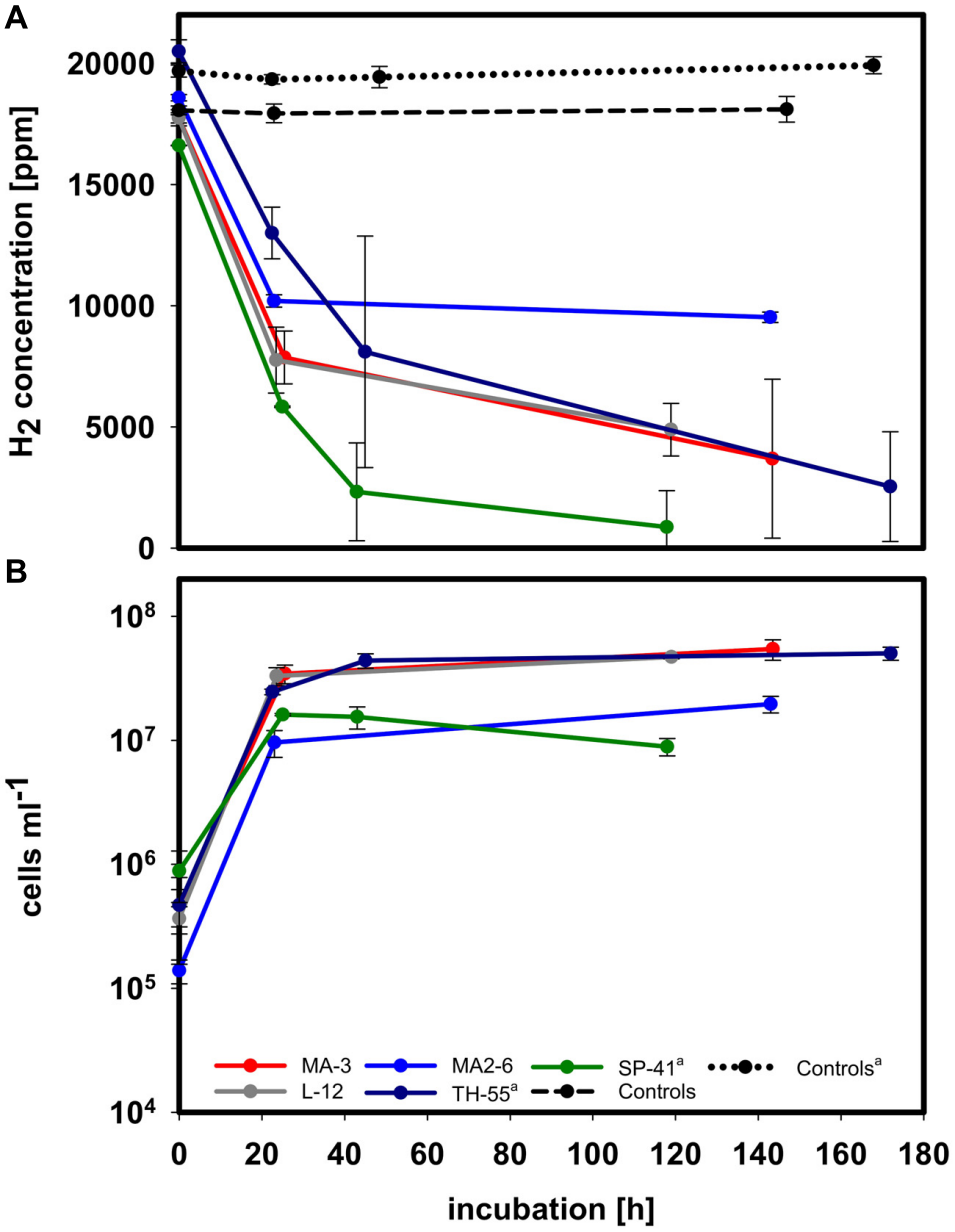
**H_2_ consumption measurements in MJ-T medium and under an atmosphere of H_2_:CO_2_:O_2_:He (2:20:1:77).** All experiments were performed at 28°C. **(A)** H_2_ concentration in the headspace of all hydrogen consuming species during incubation. **(B)** Cell density during incubation of the respective species. Exemplarily two sets of controls, non-inoculated medium treated the same way as the samples, are shown. (^a^) Data from Hansen and Perner (2015).

Except for XCL-2 and JB-B2, all other tested *Thiomicrospira* strains encoding a group 1 [NiFe]-hydrogenase (**Figure 1B**) were able to consume hydrogen and grow in our standard MJ-T medium (0.6 mM thiosulfate) with 2% hydrogen and 1% oxygen (**Figure 3**). These were: TH-55 and SP-41, as tested previously (Hansen and Perner, 2015) as well as MA-3, L-12, and MA2-6. The strains MA-3, L-12, and MA2-6 consumed most of the hydrogen during exponential growth within the first 24 h (roughly 56% for MA-3 and L-12 and 45% for MA2-6), exhibiting hydrogen consumption rates of 0.63 ± 0.1 fmol H_2_ h^-1^ cell^-1^, 0.76 ± 0.1 fmol H_2_ h^-1^ cell^-1^ and 2.1 ± 0.5 fmol H_2_ h^-1^ cell^-1^, respectively. After 24 h MA-3 and L-12, encoding a cluster II hydrogenase, continued to rapidly utilize hydrogen, where 20 and 28% of the given hydrogen was left after 144 h and 119 h, respectively. Other *Thiomicrospira* with a cluster II hydrogenase are SP-41 and TH-55. SP-41 used 35% of the given hydrogen within the first 24 h, 5% were left after 118 h, and TH-55 used 37% within 24 h and 12% were left after 172 h (Hansen and Perner, 2015). In contrast, MA2-6, encoding a cluster I hydrogenase, still had 51% of the hydrogen left in the incubation after 143 h. This distinct consumption behavior of MA2-6 was observed in two completely independent experiments (**Figure 3** and **Supplementary Figure S1**). It will be interesting to see how other cluster I hydrogenases behave and whether the cessation of hydrogen consumption at 10000 ppm is related to the type of hydrogenase and its affinity to hydrogen.

Growth experiments in MJ-T medium of the hydrogen consuming strains (**Supplementary Figure S2**) to compare maximum cell density with and without hydrogen in the incubations, demonstrated that TH-55, L-12, and MA2-6 grew significantly denser when hydrogen was present (*P*-value ≤ 0.02). MA-3 grew considerably but not significantly denser with hydrogen (*P*-value 0.058) and SP-41 did not grow denser with hydrogen, which may result from the experimental setup (see Supplementary Material and Hansen and Perner, 2015). Consequently, some of the growth in most of these *Thiomicrospira* strains appears to be related to the oxidation of hydrogen.

**FIGURE 4:**
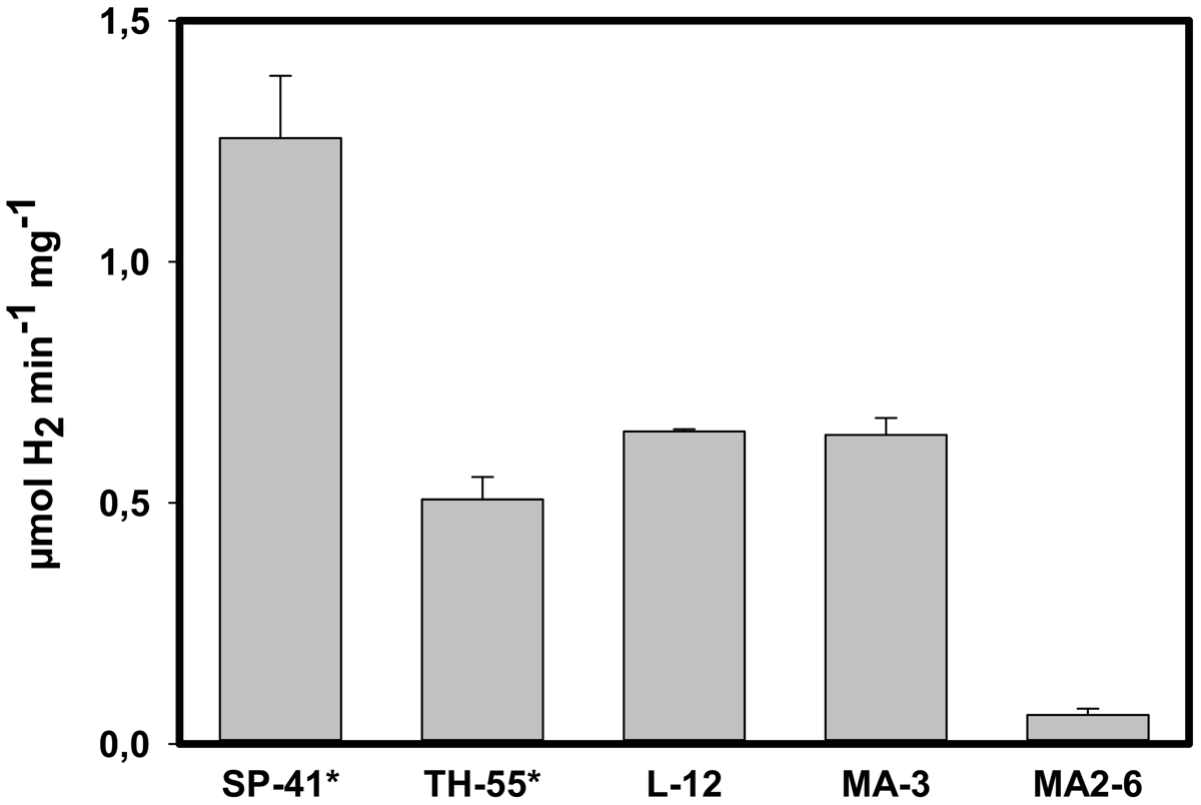
**Hydrogen uptake activity in partially purified proteins of the H_2_ consuming *Thiomicrospira* species.** The activity was located in the 30–50% ammonium sulfate fraction, i.e., solubilized membrane associated proteins precipitating between 30 and 50% ammonium sulfate saturation. ^∗^Data from Hansen and Perner (2015).

Hydrogen uptake activity was found to be in the 15–30% ammonium sulfate fraction of membrane associated proteins for all hydrogen consuming species, namely SP-41 and TH-55 (Hansen and Perner, 2015), L-12, MA-3, and also for MA2-6 (**Figure 4**): SP-41: 1.26 ± 0.13 μmol H_2_ min^-1^ mg^-1^ and TH-55: 0.51 ± 0.05 μmol H_2_ min^-1^ mg^-1^ (Hansen and Perner, 2015), L-12: 0.65 ± 0.005 μmol H_2_ min^-1^ mg^-1^, and MA-3: 0.64 ± 0.04 μmol H_2_ min^-1^ mg^-1^. The activity of the hydrogenase from MA2-6 was much lower with only 0.06 ± 0.01 μmol H_2_ min^-1^ mg^-1^. The *H. marinus’* membrane fraction exhibited roughly 0.15 μmol H_2_ min^-1^ mg^-1^ (Nishihara et al., 2001). If these activities derive from the cluster I and II group 1 [NiFe]-hydrogenases then a considerable lower hydrogen uptake activity may be a hallmark of the cluster I hydrogenase.

The strains XCL-2 and JB-B2, both encoding a cluster II hydrogenase, did not consume hydrogen when incubated in MJ-T medium with 2% hydrogen and 1% oxygen or with 1.5% hydrogen, and ∼21% oxygen (**Figure 2A**, **Supplementary Figure S3A**). Growth was not hindered under these conditions (**Figure 2B**, **Supplementary Figure S3B**). Since in the past increased hydrogen concentrations, low thiosulfate concentration, oxygen contents as well as nickel amendment has shown to influence hydrogen consumption ability in different species (Richard et al., 1999; Lenz et al., 2002; Laurinavichene et al., 2007; Palágyi-Mészáros et al., 2009; Hansen and Perner, 2016), we tested XCL-2 for hydrogen consumption under various conditions, i.e., in our standard medium MJ-T, without thiosulfate with and without added cysteine, varying thiosulfate and hydrogen and oxygen concentrations as well as growing it in TASW medium. XCL-2 did not consume hydrogen under any of these tested conditions (**Supplementary Figures S3** and **S4**, for more details see Supplementary Material results). In line with the lacking hydrogen consumption ability, neither membrane associated nor soluble proteins of XCL-2 or JB-B2 showed hydrogen uptake activity (data not shown).

### Attempts to Resolve XCL’s Hydrogen Consumption Inability

**FIGURE 5:**
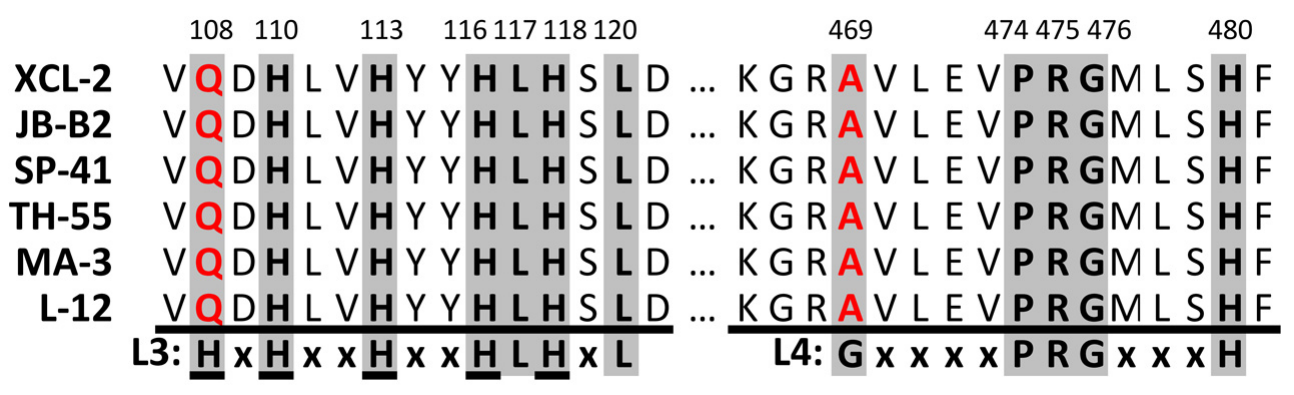
**Sequence alignment of cluster II hydrogenases.** Shown are the areas of the L3 and L4 motif and the histidine-rich region, respectively. The L3 and L4 motifs are shown in bold below the alignment, amino acids from the histidine-rich region are underlined. An *x* represents any amino acid, a gray background denotes the positions of conserved amino acids whereas amino acids marked in red indicate differences to the conserved motifs. Amino acid numbering is according to XCL-2.

Sequence information of the different *Thiomicrospira* species to compare active (SP-41, MA-3, L-12, and TH-55) and non-active (XCL-2 and JB-B2) hydrogenases is only available from the *hynL* gene. To explain the lacking hydrogen consumption ability of XCL-2 and JB-B2, we analyzed the *hynL* sequences of both strains for amino acid changes in comparison with the sequences of the active cluster II hydrogenases from SP-41, TH-55, L-12, and MA-3. We checked nine proposed and amended conserved motifs for [NiFe]-hydrogenase large subunits, in which mutations may explain the lacking hydrogen consumption ability of XCL-2 and JB-B2. The analyzed motifs were: L0 (RxEGH), L1 (RGxE and xQRxCGVCTxxH), L2 (RxCGxCxxxH and xxxDPCxACxVH), L3 (HxHxxHxxHLHxL), L4 (GxxxxPRGxxxH), L5 (DPCxxCxxH), and the histidine-rich region (HxHxxHxxHxH), (Albracht, 1994; Burgdorf et al., 2002; Szõri-Dorogházi et al., 2012; Greening et al., 2015). The L0, L1, L2, and L5 motifs are conserved in XCL-2’s and JB-B2’s hydrogenase. In contrast, in the L4 and L3 motifs (Albracht, 1994) and also in the histidine-rich region (the histidine-residues from the L3 motif; Szõri-Dorogházi et al., 2012) XCL-2 and JB-B2 each have a single amino acid exchanged (**Figure 5**). Mutations of the histidine in the L4 motif and of one leucine in the L3 motif are known to influence the O_2_ sensitivity of *Ralstonia eutropha*’s soluble hydrogenase (Burgdorf et al., 2002). In the histidine-rich region only the His110 and the His116 (numbering according to XCL-2, **Figure 5**) are highly conserved in cytoplasmic [NiFe]-hydrogenases, but in membrane bound [NiFe]-hydrogenases typically all five histidine residues are conserved (Szõri-Dorogházi et al., 2012). Mutations of the different histidine residues (His108, His110, His113, His116, and His118) have been demonstrated to affect the enzymatic activity of the *Thiocapsa roseopersicina* hydrogenase *in vivo* and *in vitro* (Szõri-Dorogházi et al., 2012). Four of these five conserved histidine residues (excluding His108) are found in the histidine-rich region of the cluster II *Thiomicrospira* group 1 [NiFe]-hydrogenases (**Figure 5**). His110 is likely part of a proton transfer pathway between the active site and the surface of the enzyme; its importance for the hydrogenase enzymatic activity was shown by different mutations up to achieving a complete loss of enzymatic activity (Szõri-Dorogházi et al., 2012). However, at the positions in the conserved motifs where XCL-2 and JB-B2 have variations, SP-41, TH-55, MA-3, and L-12 also display the same sequence variability (**Figure 5**). Hence, if the cluster II [NiFe]-hydrogenases are responsible for the hydrogen consumption ability then the exchanged amino acids are not the reason why XCL-2 and JB-B2 cannot consume hydrogen. Since from the JB-B2 hydrogenase operon only the *hynL* gene sequence is partially known, we cannot exclude that JB-B2 may have lost parts of the hydrogenase operon, e.g., maturation genes, which would explain why it cannot express a functional hydrogenase and therefore the JB-B2 *hynL* gene may be an artifact of a hydrogenase operon. Only genome sequencing could verify or falsify this hypothesis.

In case of XCL-2 the genome is available though, so we compared the gene arrangement of the hydrogenase operon (XCL-2 genes Tcr_2035 to Tcr_2043) with five operon structures from bacteria that contain the most similar *hynL* genes (*Thioalkalimicrobium microaerophilum*, *Sulfuricurvum kujiense*, *Sulfurovum* sp. AR, *Sulfurimonas autotrophica* as well as *Arcobacter nitrofigilis*; **Supplementary Figure S5**). One difference in XCL-2 compared to all other above mentioned operons is obvious: XCL-2’s operon is not interrupted by genes, which are not apparent parts of the hydrogenase machinery (operons of *S. autotrophica* and *Sulfurovum* sp. *AR* are interrupted by hypothetical genes, for which the affiliation to the hydrogenase machinery cannot be excluded with certainty). The large subunits of these strains have the L1 and L2 motifs of the prototypical [NiFe]-hydrogenases (subgroup 1b; Greening et al., 2015). All of these strains also encode for such an untypical large small subunit gene with a domain for a pyridine nucleotide-disulfide oxidoreductase as found in XCL-2. Moreover, in none of these genes (including that from XCL-2) the [Fe-S]-cluster binding sites, specific for the subgroups of group 1 [NiFe]-hydrogenases (Greening et al., 2015), appear to be conserved, implying that [Fe-S]-clusters may not be bound properly. Hydrogen utilization has been reported for, e.g., *S. kujiense* (Kodama and Watanabe, 2004). However, since *S. kujiense* encodes for different [NiFe]-hydrogenase, we cannot associate its hydrogen utilization with the XCL-2-like hydrogenase operon for certain.

We also aligned all other genes of the XCL-2 hydrogenase operon with its 10 best blastp hits (from 72 to 40% amino acid sequence identity) from different genera and checked for amino acid changes only occurring in XCL-2. The alignments revealed up to seven single amino acid differences in the genes of the hydrogenase operon of XCL-2, but none were located in regions which appear to be conserved motifs. Hence, from its sequence we did not find any explanations why XCL-2’s hydrogenase may not be functional.

XCL-2 does not encode for a cytochrome b subunit to anchor the hydrogenase in the membrane (Scott et al., 2006). Scott et al. (2006) expected XCL-2’s hydrogenase to be in the cytoplasm, because the small subunit is missing a characteristic Tat signal (important for the translocation of the enzyme into the periplasm). In Tat-mutants from *Ralstonia eutropha* active hydrogenases shifted from the membrane fraction to the soluble fraction (Bernhard et al., 2000), indicating that they were not transported into the periplasm when the Tat-machinery is turned off. If the missing Tat-signal in XCL-2 would prevent the correct localization of the hydrogenases, one would expect to find hydrogenase activity in the soluble fraction. In fact, none of the fractions were active (data not shown), indicating that no active hydrogenases were expressed by XCL-2 under the provided conditions. In contrast, the hydrogen uptake activity of SP-41 and TH-55 (Hansen and Perner, 2015) as well as of L-12 and MA-3 was located in the membrane fraction (**Figure 4**). Given that mutations in the Tat signal of the hydrogenase 2 small subunit of *Escherichia coli* suggest an influence not only for location, but also for the processing of the large subunit (Rodrigue et al., 1999), one would expect inactive hydrogenases, if this signal is missing. If the relationship between the absence of the Tat signal and processing of the large subunit is also true for XCL-2’s hydrogenase, the absences of the signal sequence may explain why XCL-2 does not appear to exhibit active hydrogenases. Unfortunately, sequence information from the small subunits of the hydrogen consuming species, i.e., SP-41, MA-3, L-12, and TH-55, for comparison with XCL-2 is not available.

In summary, we were not able to clarify for certain why experimental results for XCL-2 (and also JB-B2) are that different compared to the data known for SP-41, L-12, MA-3, and TH-55. One reason may be that XCL-2 does not express active hydrogenases under the various tested conditions here, although some of these conditions also tested for SP-41, L-12, MA-3, and TH-55 resulted in active hydrogenases produced by these strains. Another possibility is that XCL-2 was able to utilize hydrogen at some time point during its evolution, but the hydrogenase operon may have gone through various mutations (e.g., loss of cytochrome b subunit, Tat-signal and [Fe-S]-cluster binding sites) and may not be able to produce active hydrogenases anymore.

### Habitat-Specific or Species-Specific Hydrogenases

With respect to *Thiomicrospira* species hosting [NiFe]-hydrogenase genes, two general trends are visible: (i) all investigated *T. crunogena* species and close relatives have [NiFe]-hydrogenases (**Figure 1B**) and (ii) all tested *Thiomicrospira* from MAR and Eastern Pacific vents have a [NiFe]-hydrogenase (**Figure 1B**) and most of them can consume hydrogen (**Figure 3**). For *Epsilonproteobacteria* it was shown that species from hydrothermal vents share a habitat-specific set of genes, which seems to be transferred into the species independently, whereas closely related species from different habitats do not have these genes (Zhang and Sievert, 2014). However, not all hydrothermal vent *Thiomicrospira* species have hydrogenases: those from Mediterranean and Western Pacific vents appear to lack a hydrogenase (Takai et al., 2005; Hansen and Perner, 2015, Bioprojects PRJNA204054 and PRJNA234827, K. Scott) and cannot consume hydrogen (**Figure 2**). Also, what contradicts this idea on habitat-specific hydrogenase genes for *Thiomicrospira* is that JB-B2, isolated from the Wadden Sea and closely related to *T. crunogena* species (**Figure 1A**), has a *hynL* gene (**Figure 1B**) and is the only non-hydrothermal vent *Thiomicrospira* – among the tested species – that has this gene. Although, given its hydrogen consumption inability, the JB-B2 *hynL* gene may be a relic from an ancestor that colonized vents and JB-B2 is in the process of losing this trait. Genome comparisons may shed some light on such questions. One way to test whether the cluster II hydrogenase is bound to the *T. crunogena* species and/or to the geography, i.e., MAR and Eastern Pacific vents, would be to investigate whether Western Pacific *T. crunogena* species also harbor this gene. However, the only two described *T. crunogena* species from Western Pacific vents, namely 37-SI-2 and HY-62 (Petri et al., 2001; Nunoura and Takai, 2009) appear to be not available.

Biogeography can play a major role for populations. Biogeography patterns can for example be seen in vent symbiont populations of *Bathymodiolus* mussels and the shrimp *Rimicaris exoculata* (DeChaine et al., 2006; Petersen et al., 2010), in the marine *Alteromonas macleodii* strains (Ivars-Martínez et al., 2008), in *Persephonella* populations originating from deep sea hydrothermal vents (Mino et al., 2013), and in *Hyphomonas* strains from the Pacific Ocean and the Atlantic Ocean (Li et al., 2014). Thus, biogeography may strongly impact the distribution of the *hynL* genes encoded by *Thiomicrospira* species as well. Nevertheless, what contradicts this hypothesis is the random similarity between the different *hynL* genes (**Table 3**). No pattern characterizing the distribution of the cluster II [NiFe]-hydrogenase genes becomes apparent: there are identical hydrogenase sequences originating from the Eastern Pacific ridges (TH-55 and L-12) and the MAR (MA-3), but then L-12 is less similar to XCL-2 (both from the Galapagos rift) than to JB-B2 from the Wadden Sea.

**Table 3:** Similarities of the amino acid sequence of all cluster II hydrogenases.

Strain	HynL amino acid sequence similarity [%]				
	
	JB-B2	MA-3	L-12	XCL-2	TH-55
JB-B2 (Jb, Germany)					
MA-3 (TAG, MAR-N)	99.6				
L-12 (Gr, EP)	99.6	100			
XCL-2 (Gr, EP)	97.8	97.8	97.8		
TH-55 (EPR, EP)	99.6	100	100	97.8	
SP-41 (SP, MAR-S)	97.6	97.2	97.2	97.2	97.2


*Jb: Jadebusen (Wadden Sea), TAG: Trans-Atlantic Geotraverse, MAR: Mid-Atlantic ridge (North or South), Gr: Galapagos rift, EPR: East Pacific rise; EP: Eastern Pacific, SP: Sisters Peak*

All hydrogen consuming *Thiomicrospira* species so far originate from hydrothermal vents. Hence, this trait must be of advantage in these environments. It may give *Thiomicrospira* the ability to be more competitive or even outcompete other microbes inhabiting the same ecological niche at hydrothermal vents where hydrogen is available and often rapidly changing conditions dominate. It also could help *Thiomicrospira* to overcome periods of starvation from reduced sulfur compounds due to habitat-specific changing conditions. Isolation of more hydrogen oxidizing *Thiomicrospira* strains from geographically distinct habitats and generation of more hydrogenase gene sequences are needed to better understand the distribution of respective *Thiomicrospira*.

## Author Contributions

MP designed the research project, MP and MH planned the experiments. MH performed the experiments. MP and MH evaluated all data and wrote the manuscript.

## Conflict of Interest Statement

The authors declare that the research was conducted in the absence of any commercial or financial relationships that could be construed as a potential conflict of interest.
