# Supplementary material for: Hydrogenase Gene Distribution and H2 Consumption Ability within the Thiomicrospira Lineage
Source: Front Microbiol. 2016 Feb 8;7:99. doi: 10.3389/fmicb.2016.00099 (PMC4744846; doi:10.3389/fmicb.2016.00099)
Supplement: Supplementary file 1 [file Presentation_1.PDF]

Supplementary Material

Hydrogenase gene distribution and H_2 consumption ability within the *Thiomicrospira* lineage

Hansen and Perner*

***Correspondence: Mirjam Perner:** mirjam.perner@uni-hamburg.de

Supplementary Results

To investigate whether XCL-2's lack of hydrogen consumption was related to hydrogen, thiosulfate or oxygen concentrations, we tested hydrogen availability at 2% and 80%, thiosulfate at 4 mM and 40 mM and oxygen at 1% or ~21%. Nickel concentration was in all experiments 3 µM. Growth of XCL-2 with 4 mM thiosulfate in MJ-T medium, after nickel amendment with 40 mM thiosulfate in TASW medium and with high hydrogen concentration was comparable to growth in our standard medium MJ-T medium (figure S3b and figure S4). Both XCL-2 and JB-B2 grew slower when exposed to 21% oxygen but eventually reached the same cell densities as in the standard MJ-T medium (figure S3b). However, XCL-2's growth was considerably impaired when incubated only with hydrogen and no sulfur source (MJ medium) or when incubated with hydrogen and cysteine as only sulfur source (MJ-C medium) (figure S3b). Hydrogen was not consumed under any of these conditions (figure S3a and figure S4).

Since the strains *T. frisia*, Milos-T1, *T. chilensis*, *T. pelophila* and Art-3 did not grow in MJ-T medium (supplementary figure S6), we tested their growth and hydrogen consumption ability under other conditions, namely in Tp medium with amended Ni (3 µM) and Fe (30 µM), 2% hydrogen and 1% oxygen. Under these conditions only *T. frisia* grew (figure 2b and supplementary figure S7). For Milos-T1, *T. chilensis*, *T. pelophila* and Art-3 the headspace was changed to a higher oxygen concentration (~21% oxygen and 1.5% hydrogen) as stated in materials and methods part, which made them grow (figure 2b).

Supplementary Figures

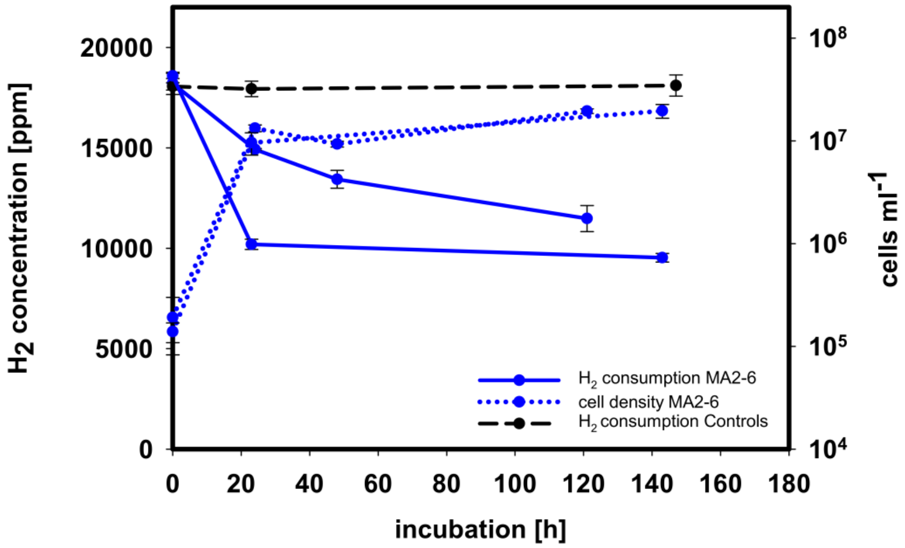

Figure S1: H_2 consumption measurements with MA2-6 in MJ-T medium under H_2:CO_2:O_2:He (2:20:1:77). H_2 concentration in the headspace during incubation is shown as blue line, cell density of MA2-6 as dotted blue line and controls, non-inoculated medium under the same conditions, as broken black line.

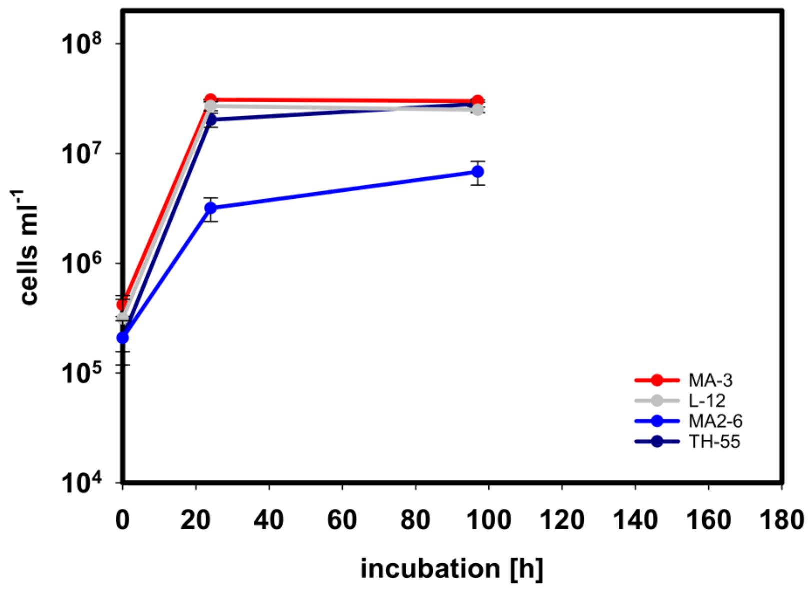

Figure S2: Growth experiments without H_2 in the headspace. H_2 consuming species were tested for growth in MJ-T medium as described for H_2 consumption measurements, but the headspace was N_2:CO_2 (80:20) and added air to give 1% O_2.

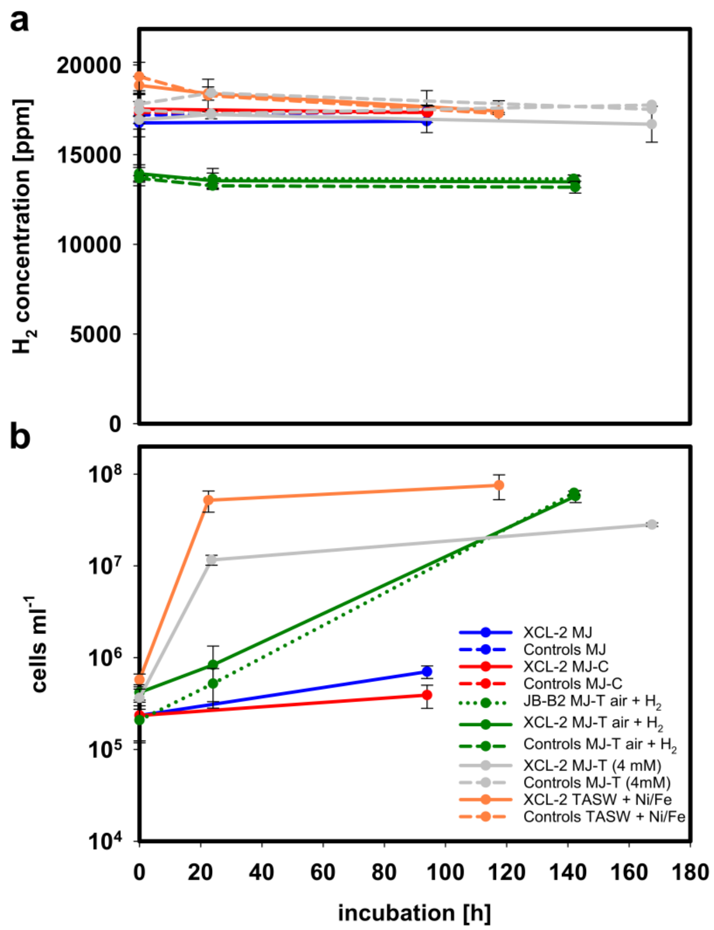

Figure S3: H_2 consumption measurements with XCL-2 and JB-B2 in different media and under different conditions. XCL-2 was tested for H_2 consumption in TASW with Ni and Fe, MJ medium, MJ-C medium and in MJ medium with 4 mM thiosulfate, all under H_2:CO_2:O_2:He (2:20:1:77). XCL-2 and JB-B2 were tested in MJ-T medium in air with added H_2 (approximately 1.5% final concentration). **(a):** H_2 concentration in the headspace during incubation. **(b):** Cell density during incubation under the different conditions. Controls of the different media and conditions are shown as broken lines in the same color as the samples of the respective condition. Controls for TASW medium were performed in duplicate.

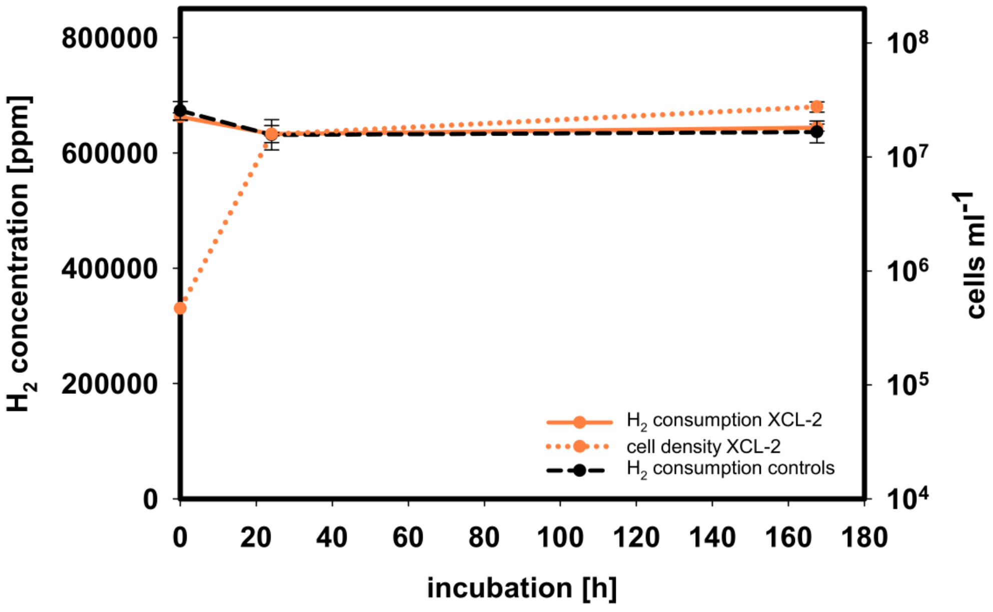

Figure S4: H_2 consumption measurements with XCL-2 in MJ-T medium under H_2:CO_2:O_2 (79:20:1). H_2 concentration in the headspace during incubation is shown as orange line, cell density of XCL-2 as dotted orange line and controls, non-inoculated medium under the same conditions, as broken black line.

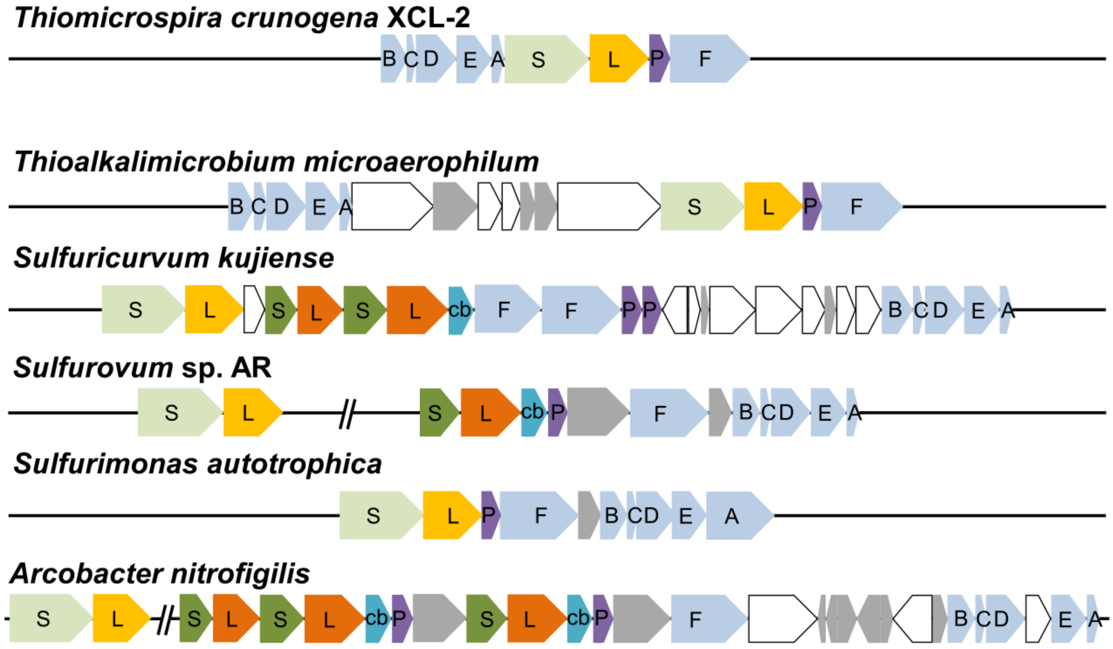

Figure S5: Hydrogenase operon arrangement of XCL-2 in comparison with the operons of *T. microaerophilum, S. kujiense, Sulfurovum sp. AR, S. autotrophica and A. nitrofigilis (Pati et al., 2010; Sikorski et al., 2010; Han et al., 2012*, Bioprojects PRJNA263055 and PRJDB84*).* The genes in the operons are color-coded: orange: large subunits (L), green: small subunits (S), light blue: Hyp-genes (A-F), cyan: cytochrome b subunit (cb), purple: hydrogenase maturation peptidase (P) and grey: hypothetical proteins. Genes not part of the hydrogenase machinery are shown in white. When more than one *hynL* and *hynS* gene is present, the genes most similar to XCL-2's have the same color as in the XCL-2 operon, other genes are shown in a different shade of the respective color.

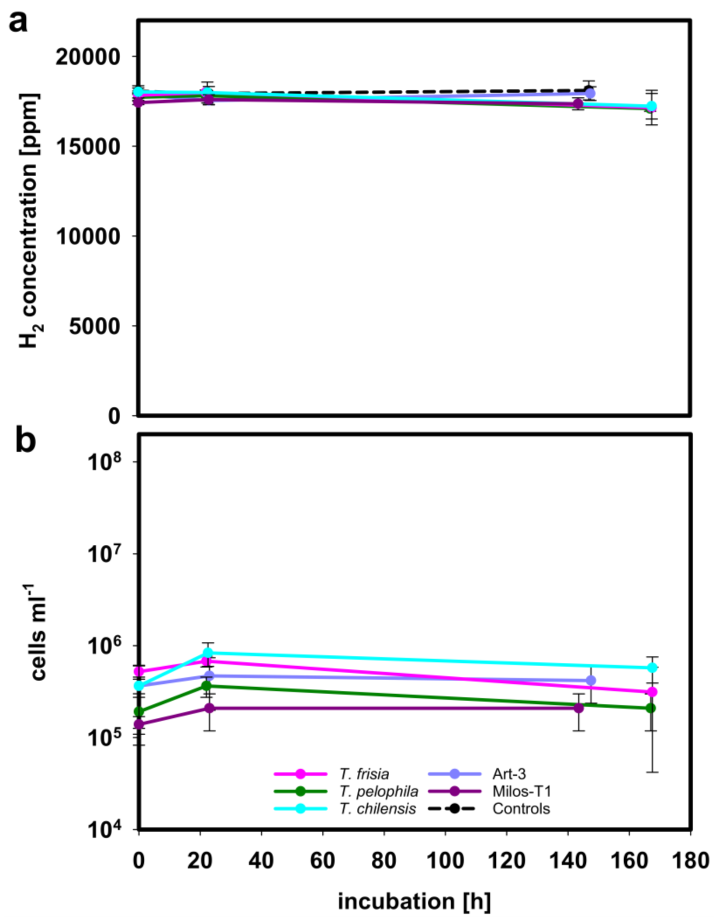

Figure S6: H_2 consumption measurements in MJ-T medium. The experiments were performed with *T. frisia*, Milos-T1, *T. chilensis*, *T. pelophila* and Art-3 under an atmosphere of H_2:CO_2:O_2:He (2:20:1:77) and at 28°C. **(a):** H_2 concentration in the headspace during incubation. **(b):** Cell density during incubation of the respective species. Exemplarily one set of controls, non-inoculated medium treated the same way as the samples, is shown as broken black line.

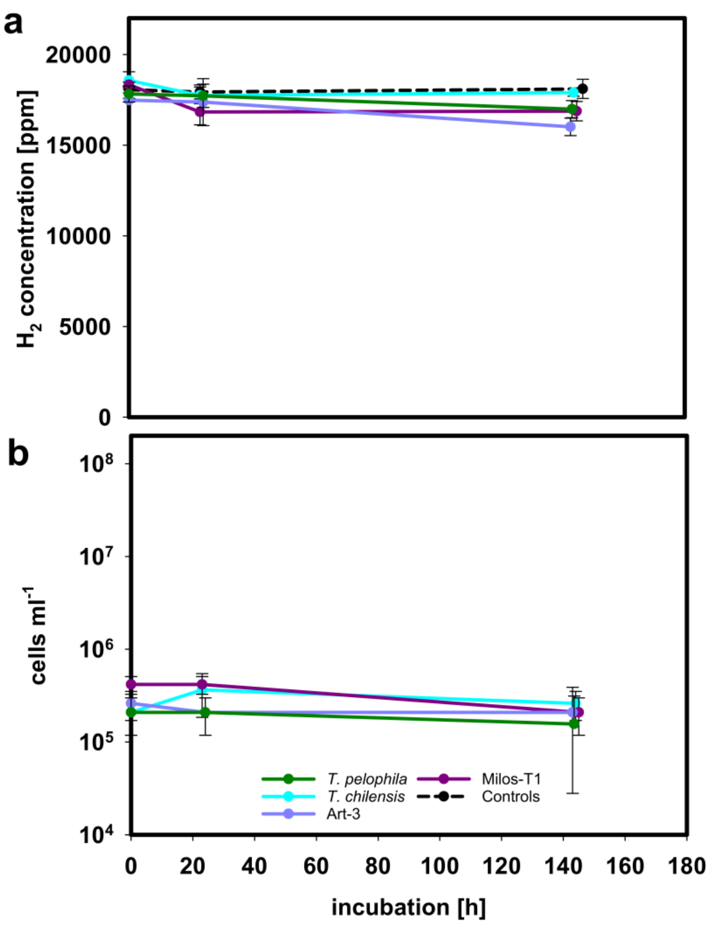

Figure S7: H_2 consumption measurements in Tp medium with Ni and Fe under H_2:CO_2:O_2:He (2:20:1:77) performed with Milos-T1, *T. chilensis*, *T. pelophila* and Art-3 at 28 °C. **(a):** H_2 concentration in the headspace during incubation. **(b):** Cell density during incubation of the respective species. Exemplarily one set of controls, non-inoculated medium treated the same way as the samples, is shown as broken black line.

References

Han, C., Kotsyurbenko, O., Chertkov, O., Held, B., Lapidus, A., Nolan, M., et al. (2012). Complete genome sequence of the sulfur compounds oxidizing chemolithoautotroph *Sulfuricurvum kujiense* type strain (YK-1(T)). *Standards in Genomic Sciences* 6**,** 94-103.

Pati, A., Gronow, S., Lapidus, A., Copeland, A., Glavina Del Rio, T., Nolan, M., et al. (2010). Complete genome sequence of *Arcobacter nitrofigilis* type strain (CI(T)). *Standards in Genomic Sciences* 2**,** 300-308.

Sikorski, J., Munk, C., Lapidus, A., Ngatchou Djao, O.D., Lucas, S., Glavina Del Rio, T., et al. (2010). Complete genome sequence of *Sulfurimonas autotrophica* type strain (OK10(T)). *Standards in Genomic Sciences* 3**,** 194-202.
